# Diagnostic Validation for Participants in the Washington State Parkinson Disease Registry

**DOI:** 10.1155/2018/3719578

**Published:** 2018-11-01

**Authors:** Hojoong M. Kim, James B. Leverenz, Daniel J. Burdick, Sindhu Srivatsal, Jennifer Pate, Shu-Ching Hu, Steven P. Millard, Marie Y. Davis, Ali Samii, Cyrus P. Zabetian

**Affiliations:** ^1^Veterans Affairs Puget Sound Health Care System, Seattle, WA, USA; ^2^Department of Neurology, University of Washington School of Medicine, Seattle, WA, USA; ^3^Lou Ruvo Center for Brain Health, Cleveland Clinic, Cleveland, OH, USA; ^4^Booth Gardner Parkinson's Care Center, EvergreenHealth Medical Center, Kirkland, WA, USA; ^5^Virginia Mason Medical Center, Seattle, WA, USA

## Abstract

**Background:**

The Washington State Parkinson Disease Registry (WPDR) was created to facilitate recruitment for Parkinson's disease (PD) research studies conducted in the Pacific Northwest. The success of registries that rely on self-report is dependent on the accuracy of the information provided by participants, particularly diagnosis.

**Objective and Methods:**

Our goal was to assess diagnostic accuracy within the WPDR cohort. We randomly selected and attempted to contact 168 of the 1,278 actively enrolled WPDR participants. Those who responded were invited to undergo an interview and neurological examination performed by a PD specialist. If an in-person assessment was not possible, we sought information collected during participation in prior research studies or from review of medical records. A diagnosis was considered “validated” if the individual met UK Parkinson's Disease Society Brain Bank (UKBB) clinical diagnostic criteria for PD.

**Results:**

Data were ascertained for 106 participants; 77 underwent an in-person assessment, 21 had data available from a prior research study, and 8 provided access to medical records. Diagnostic accuracy within the overall sample was 93.4% (95% confidence interval (86.4%, 97.1%)). Seven patients did not fulfill UKBB criteria for the following reasons: early severe autonomic involvement (*n*=3), history of neuroleptic treatment (*n*=1), presence of the Babinski sign (*n*=1), or insufficient supportive criteria (*n*=2).

**Conclusions:**

Our results indicate that studies which use the WPDR for recruitment will rarely encounter patients who are misdiagnosed. This further supports the utility of the WPDR as an effective recruitment tool for PD research in the Pacific Northwest.

## 1. Introduction

2017 marked the 200^th^ anniversary of James Parkinson's *Essay on the Shaking Palsy*, describing a neurodegenerative disorder that is known today as Parkinson's disease (PD) [1]. PD is the second most common neurodegenerative disorder, and it is estimated that the number of cases in the most populous nations will double to as much as 9.3 million by 2030 [2].

Neuroprotective therapy that delays progression has remained elusive despite advances in symptomatic treatment and an improved understanding of the genetics and pathophysiology of the disease. The average time from FDA application to approval of drugs is 12 years [3], and the time spent on patient recruitment and enrollment is a major limiting factor in this process [4]. Reducing the time required for these activities could substantially accelerate drug development. Well-characterized disease registries are one tool that can provide researchers with immediate access to a pool of prescreened individuals who are willing to participate in clinical studies [5].

To date, there are several PD registries in the US established to serve different functions. The Nebraska [6] and California PD registries [7] were established primarily as epidemiologic tools to study the incidence and prevalence of the disease and to identify potential demographic and environmental risk factors. In contrast, the PD Registry of the Muhammad Ali Parkinson Center [8] and Fox Trial Finder [9] were created to facilitate enrollment in PD-related research on a national or international scale. We established the Washington State Parkinson Disease Registry (WPDR) in 2007 with a similar intent but with a focus on patients and research studies in the Pacific Northwest [10]. Diagnostic accuracy is a challenge for any disease registry in which diagnosis is by self-report, and the lower the accuracy, the lower the overall utility of both epidemiologic and research registries. In this study, we sought to validate the accuracy of self-reported PD diagnosis among participants in the WPDR.

## 2. Methods

### 2.1. WPDR Overview

The WPDR became operational and began enrollment in 2007. All prospective participants are first screened in-person, by telephone, or with an online questionnaire to determine if they have received a diagnosis of PD from a clinician (regardless of specialty). Those who self-report a diagnosis of PD are then invited to enroll; the informed consent process is completed in-person or by telephone. At the time of enrollment, information on demographics and disease characteristics is obtained. These data include initial and current motor symptoms, age at onset and diagnosis, PD-related medication and neurosurgical history, complications of treatment, nonmotor symptoms, and family history of PD. A subset of these data is updated annually using a questionnaire that is sent and returned by mail. The WPDR database and research staff are housed at the VA Puget Sound Health Care System (VAPSHCS) in Seattle. Researchers who wish to use the WPDR for recruitment submit an application which is reviewed by a committee of five investigators. Data collected in some studies that use the WPDR for recruitment are subsequently deposited back into the WPDR database to improve the depth and quality of information available for future use.

As of April 30, 2018, 2,130 PD patients have enrolled in the WPDR and after removing participants who are now deceased or have withdrawn, 1,521 are considered “active.” Sixty-two studies have previously used or are currently using the WPDR for assistance with recruitment.

### 2.2. Validation Study: Subject Selection and Data Acquisition

The goal of this project was to estimate the proportion of patients enrolled in the WPDR who met the UK Parkinson's Disease Society Brain Bank (UKBB) clinical diagnostic criteria for PD [11] based on data obtained from a randomly selected subset of the cohort. We estimated that a sample size of between 100 and 125 was sufficient for this purpose. For these estimates, we used misdiagnosis rates of 5%, 10%, and 20% to span the positive predictive values (PPV) of the UKBB criteria reported in two clinicopathologic validation studies [12, 13]. For these misdiagnosis rates, the margin of error (half-width of the 95% confidence interval) was ±5%, ±6%, and ±8% for *n*=100, and ±4%, ±6%, and ±7% for *n*=125.

**Figure 1:**
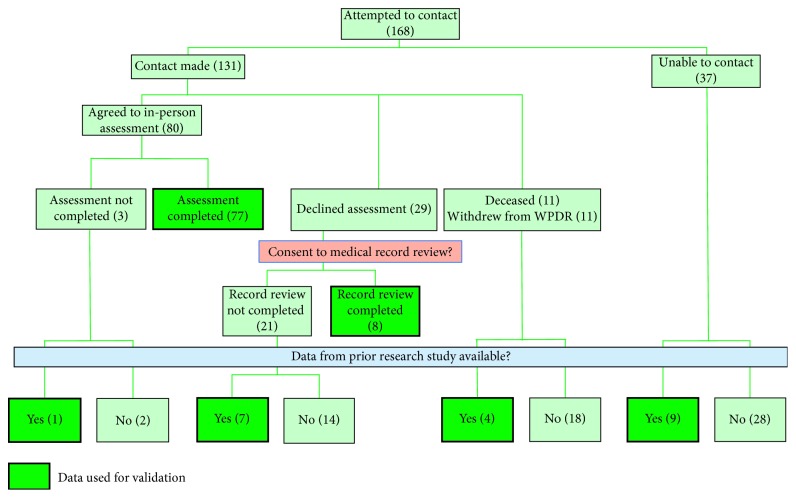
Data used for validation.

The validation study began on January 31, 2011, and at that time, a total of 1,354 patients had enrolled in the WPDR. Of those patients, we were notified of 76 deaths or withdrawals since initial entry, resulting in 1,278 active enrollments. We randomly ranked the entire active cohort from 1 to 1,278 using the sample function in the R software package and attempted to contact subjects in rank order [14]. See Figure 1 for details of the study progression. If a subject was successfully contacted, we sought permission to perform a study assessment which included a detailed neurological examination and interview. If the patient declined, we sought permission to obtain medical records from their current and/or prior neurologist(s). For the groups who were not assessed in-person, did not provide access to medical records, and could not be contacted, we searched the WPDR database to determine if they had participated in a previous study that provided suitable data back to us. Such data, if available, were used in the validation process. All study procedures were approved by the VAPSHCS Institutional Review Board, and all participants provided informed consent.

### 2.3. Validation Study: In-Person Assessments

Validation study visits were performed by a PD specialist (DJB, MYD, SCH, HMK, JBL, AS, SS, and CPZ) at our research clinic at the VAPSHCS or at the participant's place of residence. The assessment included an interview focused on PD symptoms and medications, a general neurologic examination, and the Movement Disorder Society-sponsored version of the Unified Parkinson Disease Rating Scale (MDS-UPDRS) Part III. The following information was obtained: past medical, surgical, and social history; age at onset; motor symptoms present at onset; and characteristics of tremor, rigidity, bradykinesia, and balance/gait difficulties. The general neurologic examination focused on ocular movements, sensory function, cerebellar function, general gait testing, and deep tendon reflexes.

### 2.4. Validation Study: Consensus Conferences

A core group of three PD specialists (HMK, JBL, and CPZ) attended every consensus conference, and others (DJB, MYD, SCH, AS, and SS) attended on an ad hoc basis. For each participant, a single specialist presented data available from the validation study assessment, previous studies that used the WPDR, or records from the treating neurologist. After review and discussion of the data, a consensus was reached as to whether each subject met UKBB criteria for PD. All data acquisition procedures, medical record reviews, and consensus conferences were conducted between January 31, 2011 and October 7, 2013.

## 3. Results

We initially attempted to contact the first 125 WPDR participants on the rank list and invite them to undergo an in-person assessment. Some patients could not be contacted, declined an assessment, or did not have data available from medical records or other studies. We then sought to contact an additional 43 participants in rank order until the minimum sample size was attained. In total, data from 106 patients were included in the final validation analysis (Figure 1). Of these participants, 77 were assessed in-person, 8 provided access to records from their treating neurologist (who were all movement disorder specialists), and 21 had data available from a prior research study that utilized the WPDR. This last source of data was a study on PD genetics in which a movement disorder specialist interviewed and examined each participant and based on this information determined whether the individual met UKBB criteria. We were able to reach a consensus (unanimous) decision on fulfillment of UKBB criteria for all 106 patients.

The clinical and demographic characteristics of the patients who were assessed in-person and not assessed in-person (i.e., data derived from a previous study or by medical record review) are presented in Table 1. Postural instability was more frequent in the patients who were not assessed in-person (65.5% vs. 39.0%, *p*=0.02), but otherwise there were no significant differences between the two groups.

Of the patients with data available, 99/106 (93.4%, 95% confidence interval (86.4%, 96.1%)) fulfilled UKBB criteria at consensus conference (Table 2). The reasons for not fulfilling UKBB criteria were as follows: five participants had an exclusion criterion on Step 2 (neuroleptic treatment at onset of symptoms, *n*=1; presence of the Babinski sign, *n*=1; early severe autonomic involvement, *n*=3), and two individuals had less than three supportive prospective positive criteria on Step 3. The subject with neuroleptic exposure had been diagnosed with schizophrenia over 30 years prior to the onset of asymmetric left-sided resting tremor. This individual was also judged to have excellent levodopa responsiveness. The subject with the Babinski sign had examination findings and a history that were otherwise consistent with a clinical diagnosis of PD including a longstanding response to levodopa, medication-related wearing off, and peak dose dyskinesias. Of the three subjects with early severe autonomic involvement, two were thought to meet criteria for multiple system atrophy-parkinsonism (MSA-P). Both subjects who failed UKBB Step 3 had features atypical for PD including early postural instability, only a modest response to levodopa, and in one instance, symmetric parkinsonism.

## 4. Discussion

The success of disease registries that rely on self-report is highly dependent on the accuracy of the information provided by participants, most importantly diagnosis. Using data collected from a prospective in-person assessment, supplemented with information from prior research participation and medical records, we found that a large proportion of the WPDR participants sampled (93.4%) fulfilled UKBB criteria for PD. This suggests that studies which utilize the WPDR for recruitment will only rarely encounter patients who are misdiagnosed.

We believe that there are two major factors that contributed to the high validation rate observed in our study. First, movement disorder specialists, who render PD diagnoses with greater accuracy than other practitioners [15], have cared for a greater proportion of WPDR participants than is typical for the US PD population. For example, 83.0% (88/106) of the WPDR patients successfully sampled, and 72.1% (922/1278) of the entire cohort reported that they were diagnosed or received care from a movement disorder specialist. In contrast, a recent nationwide poll conducted on behalf of the Michael J Fox Foundation found that only 45% of patient respondents had ever seen a movement disorder specialist [16]. Second, relatively few of the WPDR participants sampled were in an early stage of disease; only 24.5% (26/106) were within five years of symptom onset at the last data collection time point. Diagnostic accuracy is lowest in the early stages of PD since the assessment of some characteristics (e.g., motor progression and response to dopaminergic therapy) requires longitudinal information and features of atypical parkinsonism often emerge later in the course of the disease [17].

Of the four other large US-based PD registries of which we are aware, two (the Nebraska PD Registry [18] and the Fox Trial Finder (FTF) [19]) have performed validation studies. In the FTF study, over ten thousand members were invited to participate and 166 ultimately completed a virtual research visit via video conferencing with a neurologist specializing in PD. Though specific clinical diagnostic criteria were not used, the neurologists judged PD as the most likely diagnosis for 97% of the individuals. The Nebraska study attempted to contact 1,402 registrants and eventually interviewed and then either performed a medical record review (*n*=172) or an in-person examination (*n*=40) on a subset of patients. A movement disorder specialist used the available data to assign a “percentage probability of PD” based on global impression, and a diagnosis was considered confirmed if the probability was >50%. A PD diagnosis was validated for 82% of the patients who underwent medical record review and 77.5% of the patients who were directly examined. Comparisons between these validation efforts and ours are limited by important differences in methodology. Unlike the FTF and Nebraska studies, we examined the majority of participants in-person and used the most widely accepted clinical diagnostic criteria [11] rather than relying on a clinician's overall impression to render a diagnosis. Also, we were able to assess the validity of the diagnoses for the majority (106/168; 63.1%) of individuals who were initially targeted for recruitment in our validation study. In contrast, a much lower proportion of the target sample participated in the FTF (∼2%) and Nebraska (∼15%) studies, and thus these two studies were more susceptible to participation bias. However, it is not surprising that the validation rate of the FTF was similar to the WPDR. Both are self-selected research registries, and the participants sampled in the two studies had a similar disease duration at assessment (WPDR, 10.9 years; FTF, 8.0 years) and most had received care from a PD specialist (WPDR, 83.0%; FTF, 67.1%). The lower validation rate observed in the Nebraska study is expected since participants entered the registry as newly diagnosed cases and many of the initial diagnoses were rendered by nonneurologists.

Our study had several limitations. We were not able to perform an in-person assessment or obtain data for 36.9% (62/168) of the subjects targeted for validation for a variety of reasons (Figure 1). It is possible that the accuracy of the PD diagnosis was lower in these individuals than in those who were successfully sampled, which would bias our results. For a subset of subjects who could not be examined in-person, we used data from a prior research study to validate diagnosis. Such individuals might be more likely to have an accurate diagnosis by virtue of the fact that they previously participated in PD research. Finally, the definition of a validated case was based on clinical (UKBB) criteria alone. We selected the UKBB criteria because at the time we conducted our study, they were by far the most widely used clinical diagnostic criteria in research settings. Furthermore, the UKBB criteria have been validated in two clinicopathologic studies and were found to have PPVs of 82% [13] and 92% [12]. However, since the participants in our study did not undergo autopsy confirmation, a small proportion of the “validated” cases might have been misdiagnosed.

## 5. Conclusion

In a recent viewpoint, Dorsey and Bloem highlighted the global burden that PD poses in the coming decades, calling this the “Parkinson Pandemic” [20]. In order to meet this challenge, recruitment for clinical research must become more efficient to reduce delays in study completion. Research registries have great promise to assist in this endeavor, and since the establishment of the WPDR in 2007, sixty-two studies in the Pacific Northwest have utilized the WPDR for assistance with recruitment. Registries that rely on self-reported diagnosis of PD are limited if the diagnostic accuracy is low. Here, we have provided evidence that the vast majority of participants in the WPDR have been accurately diagnosed with PD. This underscores the value of the WPDR as an impactful resource for use by PD research studies in the Pacific Northwest.

## Figures and Tables

**Table 1:** Clinical and demographic characteristics of the study cohort.

	Assessed in-person (*n*=77)	Not assessed in-person (*n*=29)	Combined cohort (*n*=106)	*p* value^a^
MDS-UPDRS Part III, mean (SD)	37.0 (18.0)	N/A	N/A	N/A
Hoehn and Yahr scale score median (range)	2 (1–5)	N/A	N/A	N/A
Mean (SD)	2.5 (0.9)			
Bradykinesia, *n* (%)	77 (100)	29 (100)	106 (100)	1.0^b^
Rigidity, *n* (%)	73 (94.8)	29 (100)	102 (96.2)	0.57^b^
Resting tremor, *n* (%)	51 (66.2)	24 (82.8)	75 (70.8)	0.15^b^
Postural instability, *n* (%)	30 (39.0)	19 (65.5)	49 (46.2)	0.02^b^
Male, *n* (%)	59 (76.6)	20 (69.0)	79 (74.5)	0.46^b^
Disease duration, mean (SD)	11.0 (7.5)	10.7 (7.3)	10.9 (7.4)	0.89^c^
Age at assessment, mean (SD)	69.7 (8.4)	66.6 (12.3)	68.8 (9.6)	0.15^c^
Duration between enrollment and assessment (years), Mean (SD)^d^	2.7 (1.0)	N/A	N/A	N/A

^a^Comparing patients in the “assessed in-person” and “not assessed in-person” groups; ^b^Fisher's exact test; ^c^unpaired *t*-test; ^d^calculated only for “assessed in-person” group since data ascertained for the “not assessed in-person” group was sometimes derived from clinical or research visits that occurred before enrollment in the WPDR validation study.

**Table 2:** Reasons why diagnoses failed to validate.

	Assessed in-person (*n*=77)	Data from previous study (*n*=21)	Medical record review (*n*=8)	Combined cohort (*n*=106)
*UKBB criteria*				
Fulfilled, *n* (%)	71 (92.2)	20 (95.2)	8 (100)	99 (93.4)

*Reasons for not fulfilling UKBB criteria*				
Early severe autonomic involvement	2^a^	1	0	3^a^
Neuroleptic treatment at onset	1	0	0	1
Babinski sign	1	0	0	1
<3 *Step III supportive criteria*	2	0	0	2

^a^Two of these patients met criteria for MSA-P.

## Data Availability

The data used to support the findings of this study are available from the corresponding author upon request.
